# Visualization and detection of live and apoptotic cells with fluorescent carbon nanoparticles

**DOI:** 10.1186/s12951-015-0148-7

**Published:** 2015-11-21

**Authors:** Mariia Dekaliuk, Kyrylo Pyrshev, Alexander Demchenko

**Affiliations:** Laboratory of Nanobiotechnologies, Palladin Institute of Biochemistry, National Academy of Sciences of Ukraine, 9 Leontovicha Str., Kiev, Ukraine

**Keywords:** Carbon nanoparticles (CDots), Internalization, Cell imaging, Endocytosis, Apoptosis, Fixation

## Abstract

Apoptosis is a genetically encoded cell death program that involves different processes occurring on molecular and sub-cellular levels. Here we report on its new features—the increased accumulation of fluorescent carbon nanoparticles (CDots) in cells and their changed distribution within cell interior, which can witness on altered mechanisms of their translocation through the membrane. The comparative studies of living (intact) and apoptotic cells were provided with two cell lines (HeLa, Vero) using two types of fluorescent nanoparticles (“violet” and “blue” CDots). In all studied cases the images of living and apoptotic cells were different; the apoptotic cells incorporated larger number of CDots resulting in their much brighter images. These nanoparticles are distributed in cell cytoplasm, however, when the cells are fixed and treated with detergent, their nucleus is also labeled. Flow cytometry allows distinguishing the sub-populations of living and apoptotic cells in their cultures and suggests a very cheap and easy way to characterize them.

**Electronic supplementary material:**

The online version of this article (doi:10.1186/s12951-015-0148-7) contains supplementary material, which is available to authorized users.

## Background

Apoptosis (programmed cell death) is the process of great biological significance, and its study is also of practical value, primarily for the design of new anti-cancer drugs [1]. Apoptosis is known to result in development of multiple changes in cytoplasm, mitochondria and nucleus [2]. Meantime, one of the most spectacular changes on the early steps of apoptosis is the alteration of structure of cellular membrane resulting in exposure to cell surface of anionic lipids, particularly of phosphatidylserine (PS). The latter can be detected by different methods, the most popular of which is the binding of fluorescently labeled Annexin V. [3] More direct methods are also popular, for instance, based on incorporation into outer membrane leaflet of smart fluorescence dyes. [4, 5] This approach allows detection of the general effect of increase in mobility and hydration of lipid bilayer component of the membrane. Another very general feature of apoptosis is the cell shrinkage that results in membrane vesiculation leading to, formation of “blebs” and, finally, disintegration of cell into apoptotic bodies. [6].

In view of such transformation of biomembrane properties it seems logical to expect the changes of other cellular functions, particularly the ability to internalize the macromolecular and nanoscale bodies—the endocytosis. It is well established that different endocytosis pathways share one important feature: the nanoparticle entering the cell needs to be “dressed” into intracellular vesicle formed of cellular membrane. [7] In this vesicular form, the particles may follow a cascade of different trafficking steps often ending in lysosomes that are distributed in cytoplasm. It can be conceived that the cellular membrane flexibility is important in this case, and since it increases on apoptosis [8], this may influence the endocytosis process.

Nowadays the development of new methods for cell research particularly based on implementation of nanomaterials is very relevant. In this respect, our attention was attracted by the water-soluble carbon nanoparticles (CDots) that have recently emerged as a new class of fluorescence bioimaging agents. They can be formed spontaneously in one-step thermal treatment from different organic materials and are sufficiently well characterized by different structural methods [6]. Their hydrophobic core is made of pure carbon that is surrounded by polar (hydroxyl, carboxyl, carbonyl and epoxy) surface groups, which makes them highly soluble in aqueous media and allows excluding their agglomeration during the cell entry. [9] Among the advantages of these nanoparticles is their simple and green synthesis, which can be implemented in almost any laboratory from available chemicals such as sucrose, glycerol, amino acids, starch, urea, etc. [10] Latest studies have been focused on the nature of carbon dots` fluorescence [11], the emission of individual particles [12] and their potential neuromodulatory activity. [13] During past few years, scientists have shown the possibility of surface modification of these particles for further use in cell research. [14, 15] Recent studies showed that the surface-modified nanoparticles can penetrate into the apoptotic cells, shedding light on the new field of CDots application. [16, 17] Also, the ability to visualize model membranes [18] and cells [19, 20] with carbon dots was shown. Meantime, in our knowledge, there were no attempts to apply carbon nanoparticles to visualize the changes of cell functional states, such as apoptosis.

In the present communication, we report the results of our studies on two epithelial cell lines (Vero, HeLa). Both cell types were observed as live and apoptotic ones, in which the endocytosis was followed by the fluorescence of two types of CDots forming the cellular images in confocal microscope. The results show significant increase in CDots incorporation into apoptotic cells together with the change of their intracellular distribution. After the cell fixation, the difference in their distribution in cytoplasm, but also dissimilar for living and apoptotic cells, was observed. Analysis of cell population with induced apoptosis in flow cytometer allows identifying clearly the populations of living and apoptotic cells.

## Methods

### Synthesis of carbon dots

The “violet” (from β-alanine) CDots were synthesized as previously described in [11]. Specifically, 0.5 g of β-alanine was dissolved in 2 ml of distilled water in an open vessel and treated in a MW oven (600 W, 1.5 min). The obtained material was diluted with distilled water and subjected to repeated centrifugation (MiniSpin Eppendorf, 10 min, 5000*g*) with the collection of supernatant at 25 °C. To obtain an accurate concentration of the particles, the supernatant was dried to constant weight and then dissolved. The “blue” CDots were synthesized by the modified method of D.Qu [21]: 0.25 g of citric acid and 0.2 g urea were dissolved in 10 ml of distilled water. The resulting suspension was heated at 190 °C during 2 h. The obtained material was diluted with distilled water and followed by repeated centrifugation (10 min, 5000*g*) with collection of supernatant at 25 °C.

### Cell lines and culture conditions

HeLa cells and Vero cells were cultured in RPMI 140 (Sigma Aldrich) medium with 10 % of heat-inactivated fetal bovine serum (FBS, Gibco), 1 % antibiotic solution (penicillin–streptomycin, Hyclone) in a humidified incubator with 5 % CO_2_ atmosphere at 37 °C. To induce apoptosis, HeLa and Vero cells were incubated in complete medium with camptothecin during 24 h in final concentrations 5 and 25 μg/ml respectively.

### MTT assay (cell viability test)

HeLa/Vero cells were seeded at a density of 1 × 10^4^ cells per well (96-well plate) for 24 h in standard cell culture conditions. After that, the cells were incubated with “blue” or “violet” CDots at final concentrations 16 and 75 μg/ml respectively during 1 and 24 h. After incubation, the medium was removed and replaced with 100 µL of fresh culture medium. For control groups of HeLa/Vero cells the same procedure was provided without incubation with CDots. To each well, 10 µL of 3-(4,5-dimethylthiazol-2-yl)-2,5-diphenyltetrazolium bromide, MTT (Sigma Aldrich) (10 mM) was added, which followed by incubation at 37° C for 4 h. After labeling the cells with MTT, the entire medium was removed and 100 µL of DMSO was added to each well and the cell suspension was mixed thoroughly with the pipette. Then the cells were incubated at 37 °C for 10 min and mixed again. The amount of formazan formation was controlled by absorbance at 540 nm.

### Spectrofluorimetry

HeLa cells and Vero cells were seeded into 24-well plate in concentration 2.5 × 10^5^ cells per well. For staining procedures the “blue” or “violet” CDots in PBS were added to the cells at final concentrations 16 and 75 μg/ml respectively. Control groups of live and apoptotic cells were not exposed to incubation with carbon dots. After 1 h of staining, the medium was removed, and the cells were washed twice with PBS. Fuorescence spectra were recorded with spectrofluorimeter QuantaMaster (Photon Technology International).

### Confocal microscopy

Confocal microscopy experiments were performed by using Carl Zeiss LSM 510META instrument, with objective Plan-Apochromat 63x/1.4 Oil. The excitation was provided by 405 nm diode laser. The samples were located in humidified thermostated box at 37 °C.

#### *Live cells imaging*

The cells (3 × 10^4^) were grown on the Ø25 mm glass coverslips and then incubated with “blue” or “violet” CDots at final concentrations 16 and 75 μg/ml respectively. After 1 h of staining procedures, the medium was removed, and the cells were washed two times with Ringer’s solution containing (in mMs): 125 NaCl, 5 KCl, 1 MgSO_4_, and 32 HEPES/NaOH (pH 7.4), 5 glucose, and 1 CaCl_2_. The coverslips with cells were mounted in Attofluor cell chambers (Life Technologies).

#### *Fixed cells imaging*

Cells (3 × 10^4^) were grown on the Ø15 mm glass coverslips and then fixed with 5 % paraformaldehyde for 10 min. Afterwards, the cells were washed two times with PBS and then treated by 0.5 % Triton- × 100 for 5 min and briefly washed with PBS. The cells were incubated with blue” and “violet” C-dots at final concentrations 16 and 75 μg/ml respectively. After 1 h, the medium was removed, and the cells were washed two times with PBS. Coverslips were mounted in Moviol-DABCO medium *(C. Favara, 2003, CLA)* and stored at 4 °C. The microscopy studies were performed at room temperature.

### Flow cytometry

Measurements were performed with Beckman Coulter Epic XL flow cytometer using the blue (488 nm) excitation laser line. Emission was collected with Fl1 channel (“FITC”). Additionally the forward and side scattered light was measured for each sample with the respective detection channels. To get sufficiently high signal, 2 × 10^4^ events were counted per sample. Obtained data was analyzed with FCS express software.

## Results and discussion

In this research we applied CDots that have been described and used in our recent studies. [11–13] The “violet” CDots are the first type, produced from β-alanine with a maximum excitation and emission at 350 and 440 nm respectively. The second type, the “blue” CDots, was fabricated from citric acid and urea, with a peak of excitation at 405 nm and emission spectrum in a broad range 420–650 nm. (Additional file 1: Figure S1) The synthesized nanoparticles carry weak negative charge that was detected by horizontal gel electrophoresis with 1.5 % agarose gel. The size which is less than 10 nm was measured using zeta sizer and described earlier. [11].

To optimize the fluorescence response of carbon dots after penetration into the cells, the latter were incubated with “blue” CDots at various concentrations for 1 hour. It was noted that the CDots are accumulated in the cell in a concentration-dependent manner, which was evidenced by an increase of intensity of the fluorescence spectra. (Additional file 1: Figure S2) Most probably, they enter into the cells by caveolae-mediated endocytosis. [7].

Endocytosis is a concentration-dependent process, which does not scale linearly for high nanoparticle concentrations. According to the results of our preliminary studies the concentration range 4–24 µg/ml was selected for these experiments, so that higher concentrations were avoided. The same experiment for “violet” CDots with the concentration range 25–100 µg/ml was performed. For this type of CDots we used higher concentrations due to the fact that they display lower intensity of fluorescence than “blue” CDots. We noted that in a short incubation time (1 h) both types of CDots penetrate into the cells in optimal quantities allowing their visualization. It is important that the CDots when penetrate into the cells do not lose their fluorescence properties and can be excited in the common blue-green spectral range.

To determine the cell viability before and after incubation with CDots, the MTT test was performed for both cell lines. (Additional file 1: Figure 3). During experiment, the amount of yellow tetrazolium MTT is reduced by metabolically active cells, in particular by the action of dehydrogenase enzymes, and the formation of purple formazan can be observed by spectrophotometric measurements. [22] We noted that “blue” CDots do not produce significant effect on cell viability after incubation for 1 or 24 h. The scatter of the values fitted permissible experimental error. However, “violet” CDots display more significant influence on Hela cells, so that their viability decreased by 14.4 % after incubation for 24 h. For the Vero cells, the 17 % reduction of metabolic activity was observed after 1 hour of incubation, and for longer incubation (24 h) the decrease was more than 30 %. These results indicate a possible decrease of the number of viable cells in culture, in particular the cells with the ability to divide. These calculations were performed in comparison with control group that was not subjected by incubation with CDots.

To investigate the possibility of cytotoxicity effect of carbon nanoparticles on studied cells, the experiment with long-term incubation of HeLa cell line with CDots during 48 h was provided. The measurements of fluorescence and cell counts were performed with flow cytometry. In this way, it was demonstrated that incorporated CDots do not influence on the quantity of apoptotic cells, resulting in their similar values with the control group (Additional file 1: Figure S4).

**Fig. 1:**
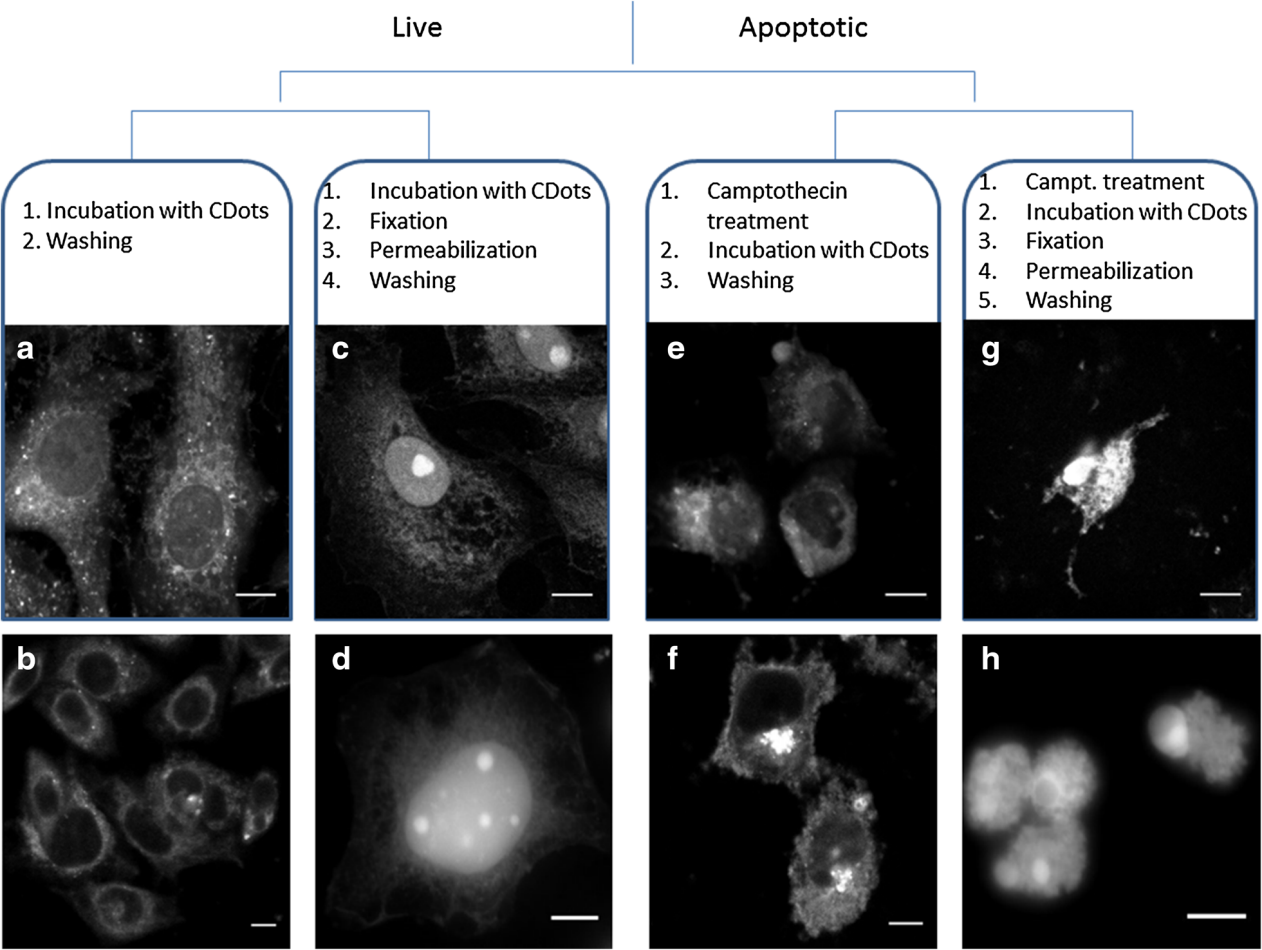
Confocal imaging of CDots labeled Vero cells (*upper row*) and HeLa cells (*down row*), excitation 405 nm. *Scale bar* is 10 µm. **a**, **b** living cells; **c**, **d** living fixed cells; **e**, **f** apoptotic cells; **g**, **h** apoptotic fixed cells

Microscopy data obtained with HeLa and Vero cells stained with CDots showed quite similar results indicating that the nanoparticles penetrate into the live cells by endocytosis. This was evidenced by the specific pattern of cell labeling, namely the appearance of vesicles (bright spots) in the cytosol and lysosomes. (Figure 1a, b) Also, due to the small size, the carbon dots can penetrate in small numbers into the nucleus through the pores in nuclear membrane. Previously, the caveolin-mediated endocytic penetration effect into the cells was studied in more details for other types of nanoparticles, particularly for small-size (3–10 nm) quantum dots and gold nanoclusters [23]. Based on the results of our experiments, we believe that we observe the process similar to that described for these small sized nanoparticles.

The possibility of CDots application as the staining agents for fixed samples represents considerable interest for cellular biology due to new visualization capabilities, such as labeling of the nucleus. For this purpose, the cell fixation with paraformaldehyde followed by treatment with Triton-X100 for further gaining of plasma membrane permeability [24] was performed (see “Methods”). These treatments should allow an easier penetration of nanoparticles inside the cells. Also indeed, confocal images showed significant extent of staining of the nucleus and intracellular vesicles at the same time. This process is probably caused instead of endocytosis by increased permeabilization of the cell membrane, and we observe that the smallest particles penetrate into the cell independently (not in vesicles) and directly through the nuclear membrane pores into the nucleus (Fig. 1c, d). Previously, it was shown that the fixed cells can also form vesicles incorporating the nanoparticles, but the majority of them did not bind directly to caveolae [25]. Visually similar results—the formation of vesicles in the cytoplasm—were obtained (Additional file 1: Figure S5), but the mechanism of their formation in fixed cells may be different.

Based on these findings we focused our efforts on characterizing the patterns of distribution of internalized CDots in apoptotic cells. Existing literature data did not allow us to predict the direction of these changes. From one side, the cell membrane on apoptosis becomes stiffer due to cell shrinking and expelling phospholipids with unsaturated acylic chains into the liquid-disordered domains to develop into blebs [8]. On the other side, the loss of transmembrane asymmetry and sphingomyelin hydrolysis into ceramide decreases the lipid order and this may favor internalization of nanoparticles, as it was observed for the permeability to ions, organic dyes and release of nucleotides [1]. Analysis of obtained images demonstrates that the CDots penetrate easier into apoptotic cells and are localized not only in lysosomes, but also in the cytosol, in significant numbers. These data witnesses not only for their increased penetration but also for their altered distribution within the cells (Fig. 1e, f).

Remarkable is the observed concentration of fluorescent CDots within the vesicles formed on the surface of apoptotic membrane that are known as blebs [1]. It can be suggested that the structure of these selected membrane sites possessing increased flexibility favors incorporation of studied nanoparticles. All that makes apoptotic cells stained with CDots visually different from that of live cells.

Can we relate observed apoptosis-related differences with the changed properties of cell membrane only? In this regard it could be natural to provide comparative studies of permeabilized fixed cells, in which the penetration of nanoparticles is greatly increased. As we observe from the data presented in Fig. 1, apoptotic cells demonstrate similar distribution of CDots compared to fixed native cells. Though the nanoparticles penetrate into the cells in larger quantities, they tend to be accumulated in cell nucleus. The early stages of the nucleus fragmentation were observed for some cells in both cell lines (Fig. 1g, h).

**Fig. 2:**
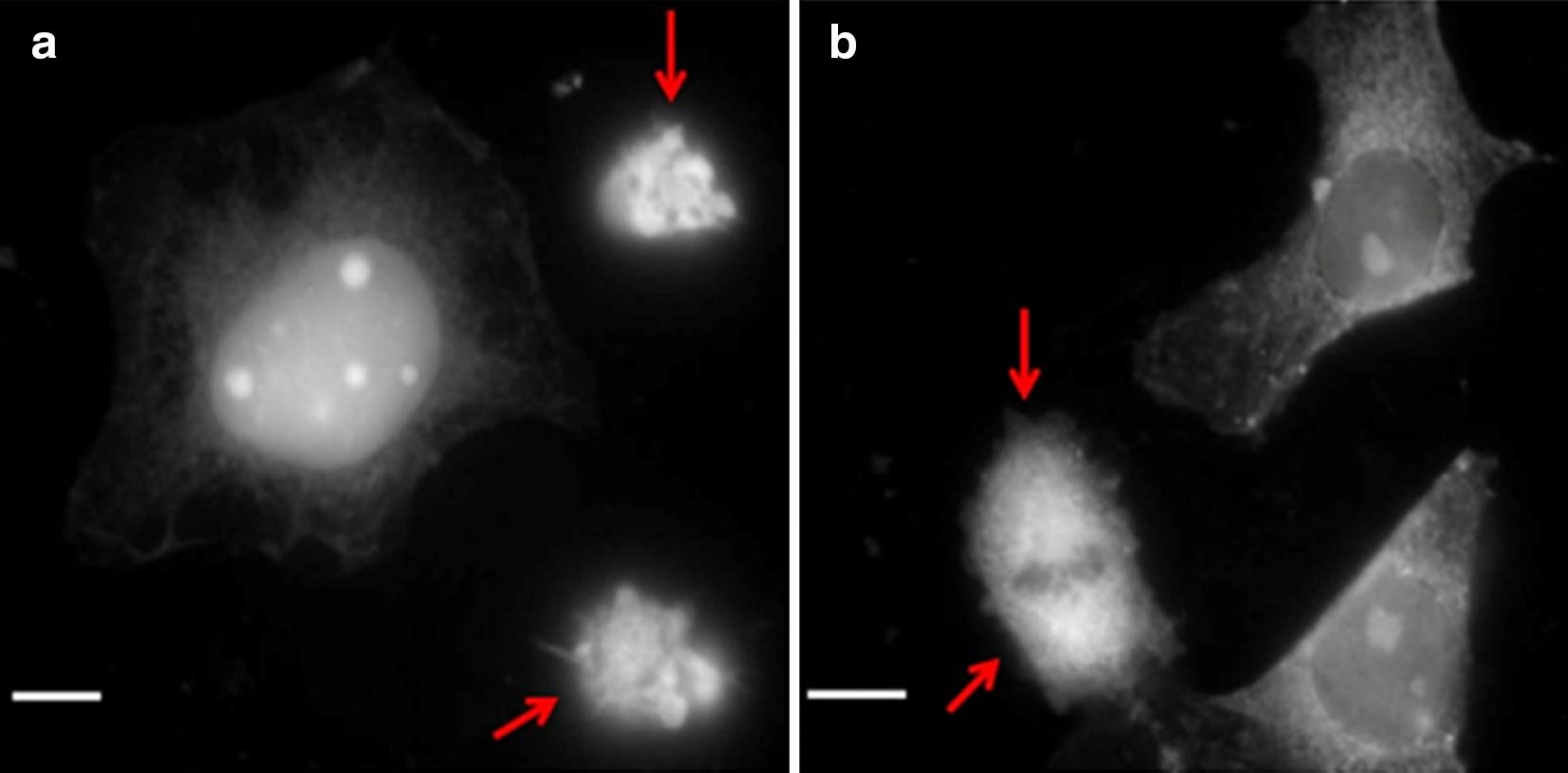
Simultaneous visualization of native and apoptotic cells (marked with *red arrows*) in a fixed sample by Violet CDots. **a** HeLa cells and **b** Vero cells. Excitation 405 nm. *Scale bar* 10 µm

Thus, being stained with CDots, live and apoptotic cells demonstrate striking differences in morphology that can be observed from the images. In line with the literature data [2] the living cell possesses larger size and well-distinguishable internal structure. In contrast, apoptotic cells are smaller and demonstrate the presence of brightly contrasting vesicles on the surface. In the same preparation both living and apoptotic cells can be found, so that they can be fixed and labeled simultaneously. An example of such images is presented in Fig. 2. Analysis of the image data on coverslips with simultaneous presence of native and apoptotic cells shows significant differences in the CDots fluorescence intensity and their distribution for these cells types. Thus, if fixed cells are compared then in contrast to intact cells the fluorescence intensity of camptothecin-treated Hela and Vero cells increased by 14 and 26 % respectively.

**Fig. 3:**
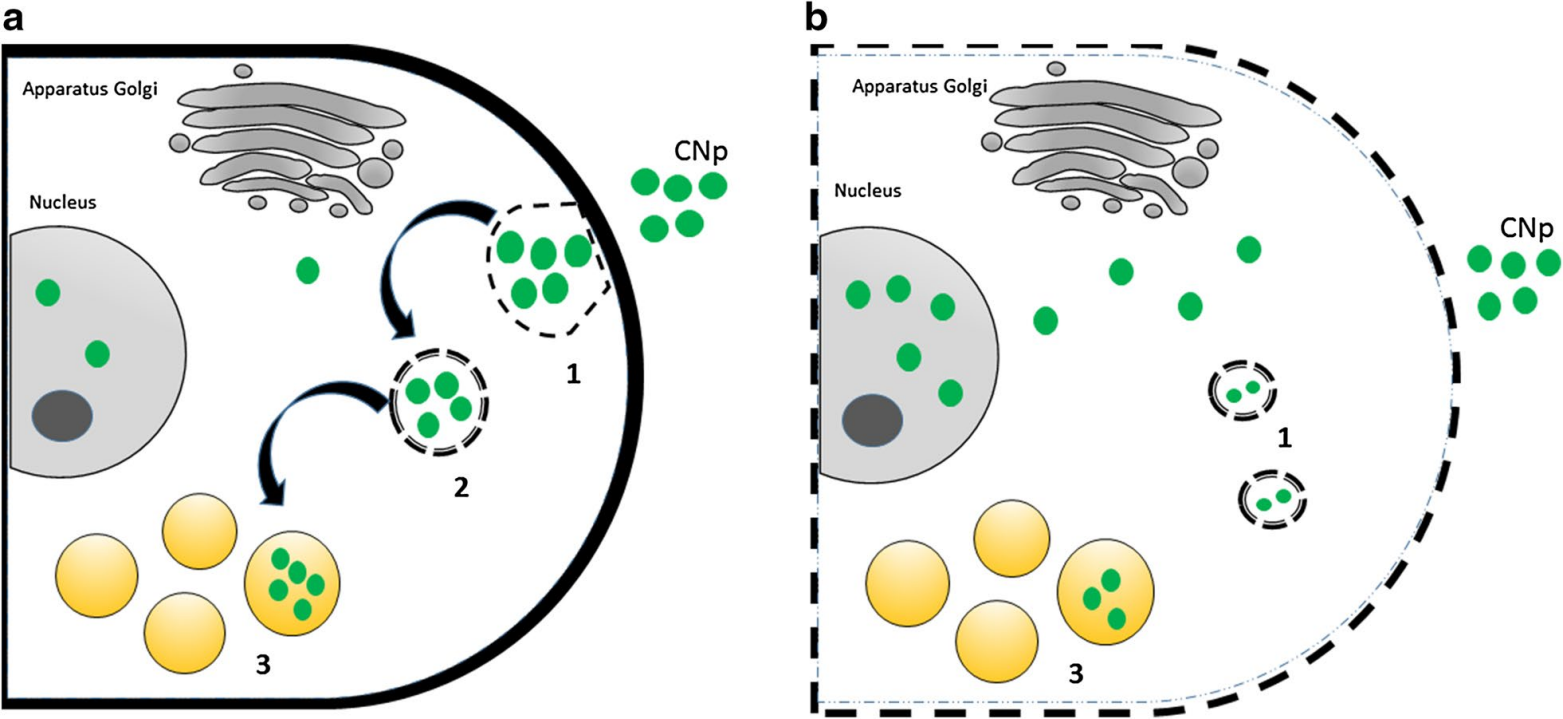
Schematic representation of cellular uptake of CDots without (**a**) and with additional cell permeabilization by Triton-x100 (**b**). *1*,*2*—vesicles, *3*—lysosomes

Schematically, the process of CDots penetration into live and apoptotic cells is visualized in Fig. 3. In line with our experimental data it can be suggested that the penetration of nanoparticles is realized for live and apoptotic cells as the process of endocytosis occurring in native conditions, so that their increased penetration can be attributed to the properties of cell membrane. Therefore their changed distribution within the cell should reflect the alteration of cell machinery. In contrast, for fixed cells a spontaneous diffusion of CDots takes place through membrane pores that allows their distribution in both cytoplasm and nucleus. Labeling of nucleus allows visualizing its condensation and fragmentation, which is known as an important step in execution of apoptosis. [2].

**Fig. 4:**
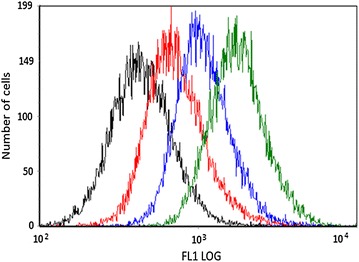
The histogram representing relative fluorescence intensity (F1LOG): autoflourescence of living cells (*black*), autofluorescence of apoptotic cells (*red*), living cells stained with *violet* CDots (*blue*), apoptotic cells stained with blue CDots (*green*)

To describe the difference in CDots distribution between living and apoptotic cells the flow cytometry was applied. The autofluorescence of living and apoptotic cells was measured before incubation (control groups), and after incubation with the two types of CDots. As we expected, the increase of fluorescence intensity of the HeLa cells was observed after treatment with these carbon nanoparticles, and the sub-population of apoptotic cells demonstrates significantly higher values of fluorescence than the intact cells (Fig. 4). To quantify the increase of the fluorescence signal the flow cytometry data was normalized as the increment of CDots signal of incubated cells to the values of untreated and unstained cells. As the result, we demonstrated increase of “blue” and “violet” signal in apopototic cells in more than 3,5 and 2,5 times in comparison to unstained cells, respectively; and in over 2 times for both types of CDots comparing to respective stained intact cells (Additional file 1: Figure S6). These data fully confirm the results obtained in microscopy experiments: the apoptotic cells accumulate much larger number of nanoparticles than the living cells.

**Fig. 5:**
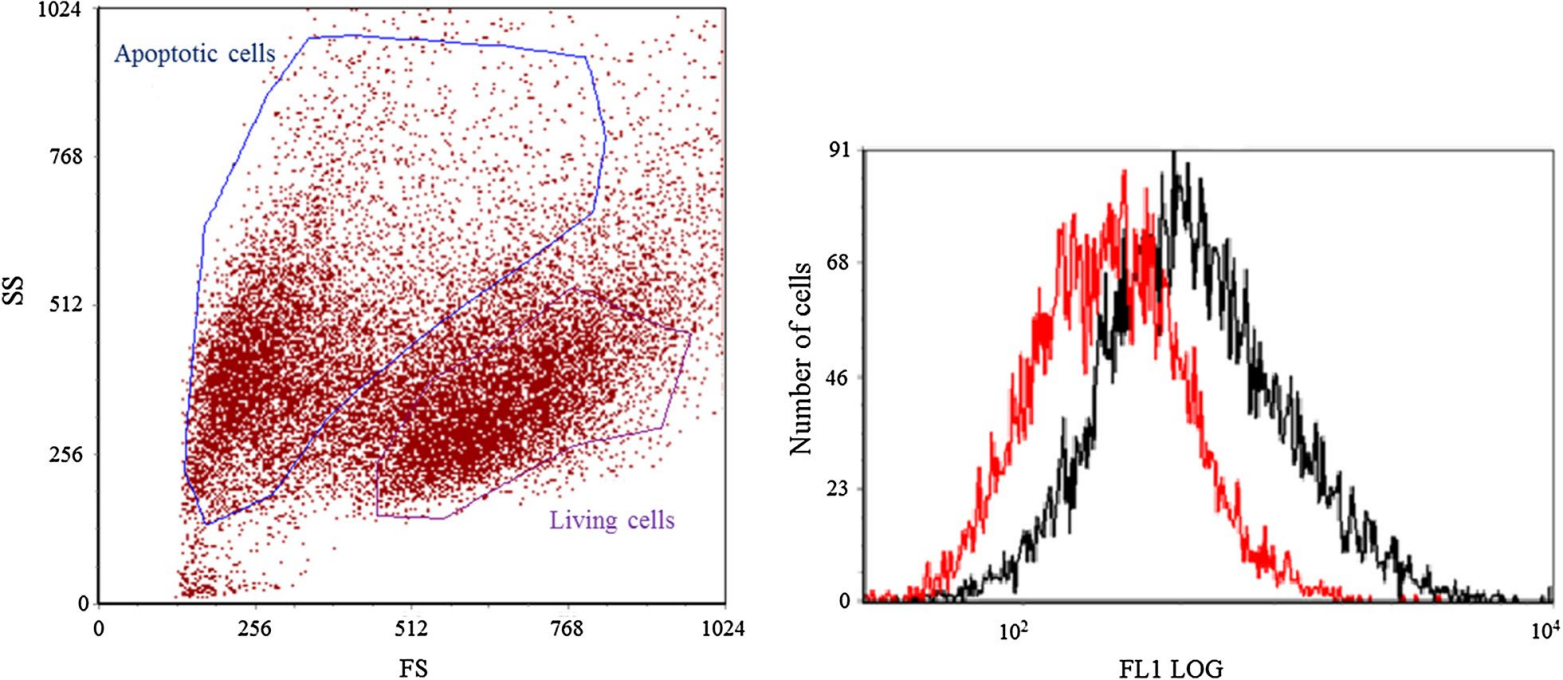
The *dot-plot* represents the distribution of camptothecin-treated HeLa cells (*left*) and histogram representing relative *CDots* fluorescence intensity for living and apoptotic cells (*right*). The cells with smaller size and higher side scattering signal were gated as apoptotic cells (*black*), the cells with larger size and lower side scattering signal were gated as living cells (*red*)

To establish the difference of fluorescence intensities of living and apoptotic subpopulations in one sample the next step of our work has been made. According to the cells’ morphology measured by the flow cytometer, the apoptotic and vital cells were gated [26] in one sample and their distribution represented as CDots fluorescence intensity histogram (Fig. 5). The sample of camptothecin-treated cells was measured to distinguish the difference in ratios of forward to side scattered light. Then according to the flow cytometry data the areas were selected similar to the cells stained with GFP-labeled Annexin V (Additional file 1: Figure S7). For these areas the histograms of CDots fluorescence intensity were plotted. Thus, we detected the difference in recorded intensities, due to specificity in accumulation of CDots inside the living and apoptotic cells, avoiding the artifacts based on the possible variations of the cells/CDots concentration ratios in different samples.

## Conclusions

In our study, we present the new approach for imaging of the apoptotic cells with fluorescent nanoparticles and demonstrate the efficient use of their new type—the CDots. The enhanced imaging possibilities of different types of nanoparticles were recognized in other recent studies [27, 28], however, the new carbon nanomaterials offer their clear advantages. Their distinctive features, such as high brightness, small sizes, high biocompatibility, small negative charge on the surface and very simple methods of their preparation present a good alternative to other nanoscale materials. We can also note that carbon dots obtained from different precursors and with different fluorescent characteristics have the same effect in the cell imaging and labeling.

With these tools we demonstrate that both living and apoptotic cells can be easily visualized. The CDots uptake occurs probably by endocytosis, which allows to accumulate in apoptotic cells much larger nanoparticles number. Using the different methods of sample preparation, they show the ability for labeling various structural compartments of the cell. For living cells there are the intracellular vesicles and lysosomes. In contrast, in fixed cells the nucleus is labeled preferentially.

The characteristic morphological signs of apoptosis (cellular shrinkage, membrane blebbing) can be easily observed in the non-fixed cell preparations. Remarkably, the blebs accumulate strongly increased amount of CDots. The studies of permeabilized fixed cells allow observation of nuclear condensation and fragmentation that also occurs on apoptosis.

The fact that apoptotic cells accumulate strongly increased amount of CDots can be efficiently used in flow cytometry for characterizing the cell populations regarding the relative amount of apoptotic cells in different experimental conditions. The application of such cheap and easily accessible nanoparticles provides more opportunities to simplify the popular methods of cell labeling and detection.

## Additional file

10.1186/s12951-015-0148-7 Fluorometry and flow cytometry measurements.
