# Supplementary material for: Visualization and detection of live and apoptotic cells with fluorescent carbon nanoparticles
Source: J Nanobiotechnology. 2015 Nov 21;13:86. doi: 10.1186/s12951-015-0148-7 (PMC4654871; doi:10.1186/s12951-015-0148-7)
Supplement: Supplementary file 1 — 10.1186/s12951-015-0148-7 Fluorometry and flow cytometry measurements. [file 12951_2015_148_MOESM1_ESM.docx]

**Visualization and Detection of Live and Apoptotic Cells with Fluorescent Carbon Nanoparticles**

**Mariia Dekaliuk, Kyrylo Pyrshev, Alexander Demchenko**

**Supporting materials**


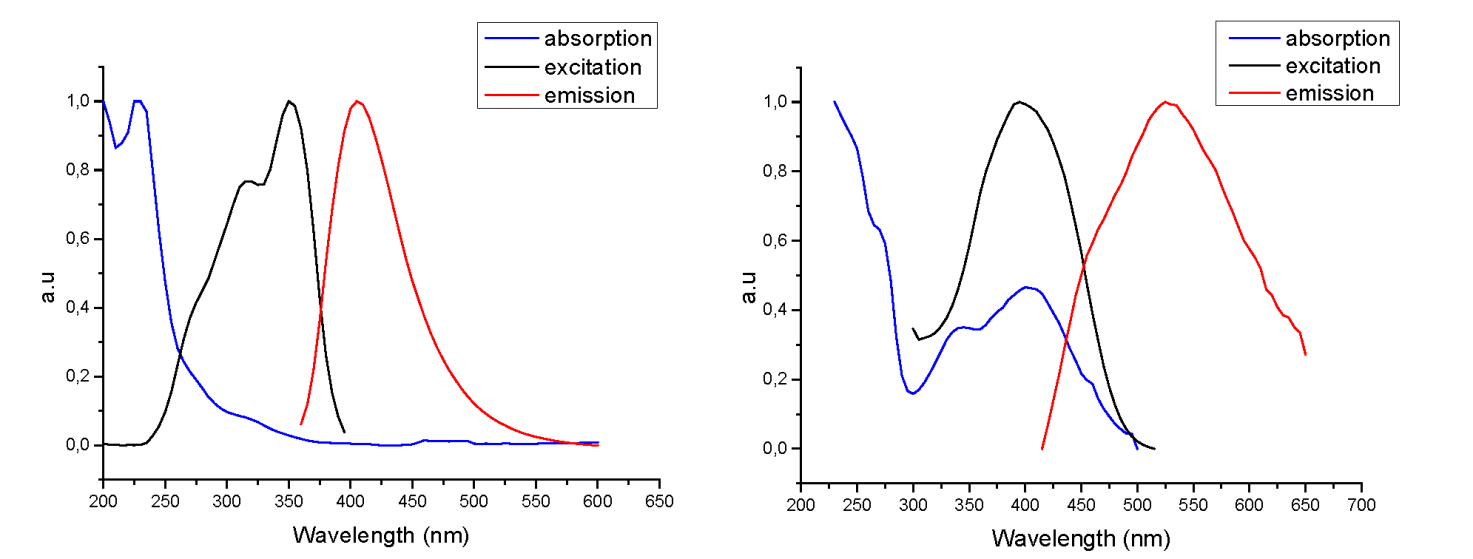


*SI Fig 1. Absorbance and fluorescence spectra of violet (left) and blue (right) carbon dots.*


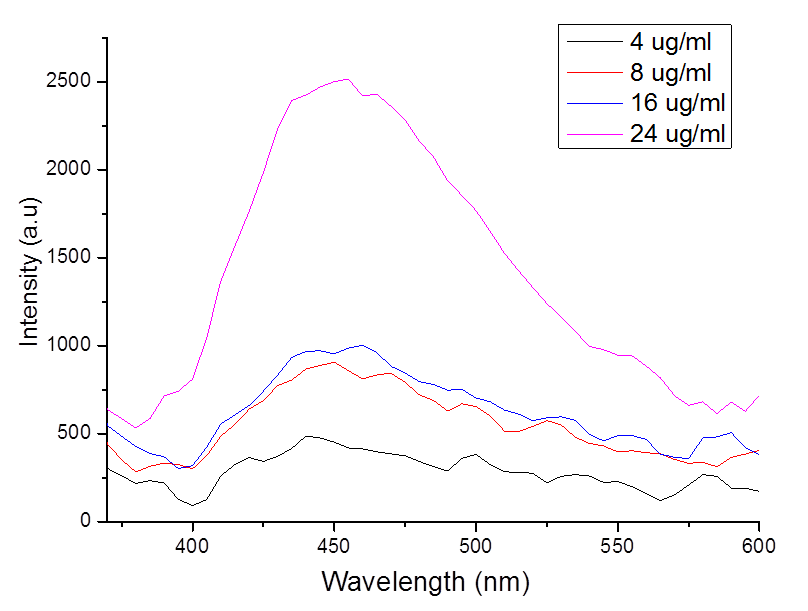


*SI Fig 2. The change in fluorescence spectra after incubation of HeLa cells (live) with “blue” CDots during 1 hour in different concentrations (autofluorescence substracted).*

*
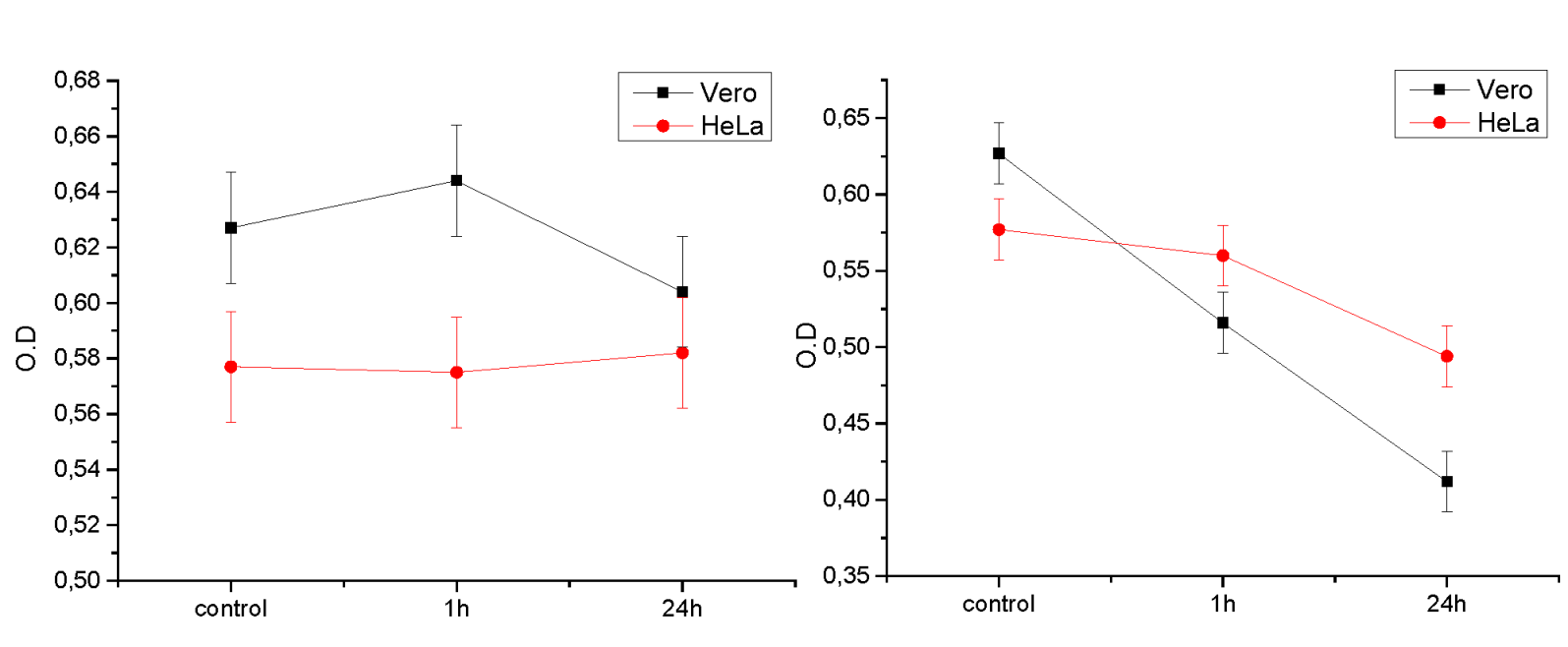
 SI Fig.3 Relationship between MTT formazan production (O.D) and time of cell incubation with blue (left) and violet (right) CDots.*


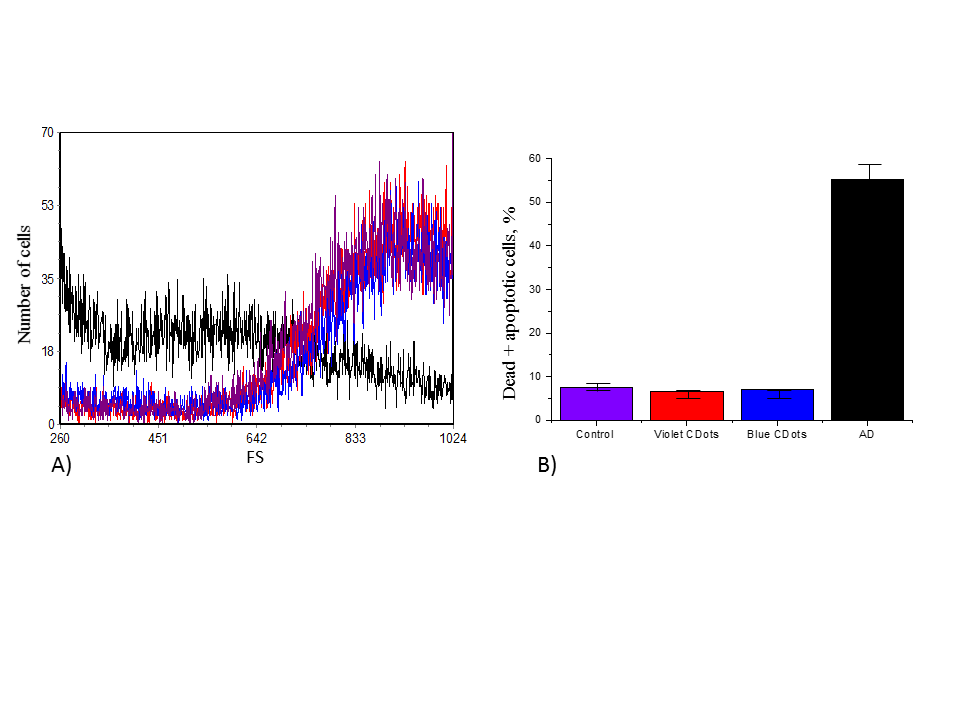


*SI Fig.4 Relative size (FS, Forward scattered light) distribution of HeLa cells (A) after incubation with Violet CDots (red), Blue CDots (blue) and Actinomycin D, AD (black) during 48 hours; control group (violet). The number of dead cells in the sample after 48 hours of incubation with the carbon dots (B). Measurements were performed by flow cytometry method.*

*
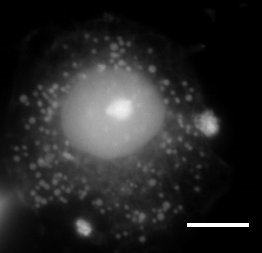
*

*SI Fig.5 The fixed Vero cell incubated with “blue” CDots. Scale bar is 10 µm.*

*
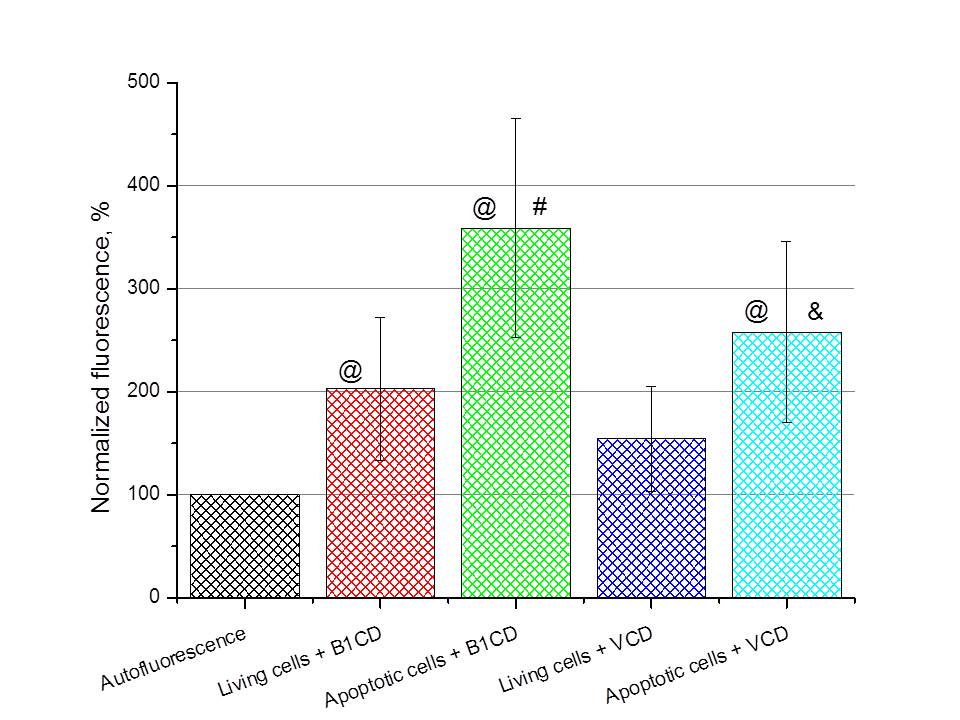
*

*SI Fig.6 The increase of the fluorescence signal in living and apoptotic HeLa cells in comparison to the values of untreated and unstained cells (autofluorescence). Apoptosis was induced with camptothecin. Given are the mean±s.d. from three independent experiments. B1CD – “blue” CDots, VCD – “violet” CDots.*

*@ p<0,05 according to the autofluorescence signal;*

*# p<0,05 according to B1CD-stained living cells*

*& p<0,05according to VCD-stained living cells*

*
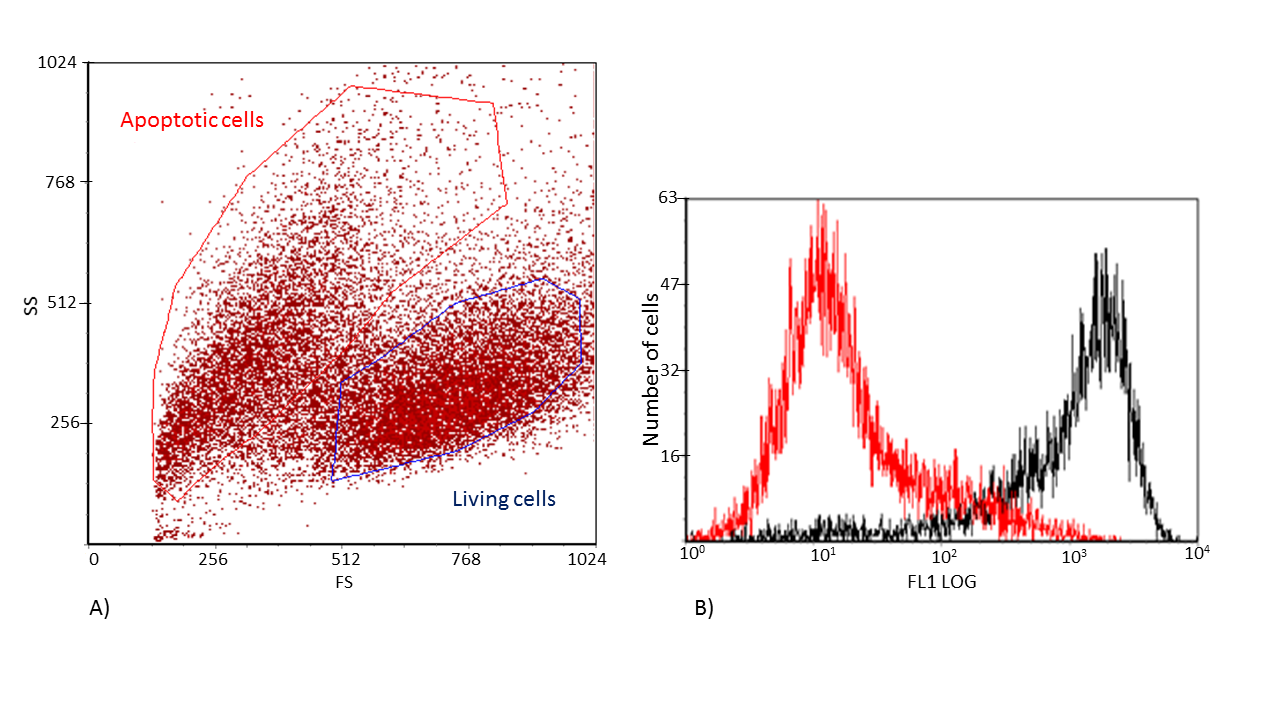
*

*SI Fig. 7 The density dot-plot represents distribution of camptothecin-treated HeLa cells. The cells with lower size and higher side scattering were gated as apoptotic cells (black), the cells with higher size and lower side scattering were gated as living cells (red). Histogram represent relative GFP-Annexin V fluorescence intensity for living and apoptotic cells. SS – side scattered light.*
